# Maternal plasma sequencing: a powerful tool towards fetal whole genome recovery

**DOI:** 10.1186/1741-7015-11-56

**Published:** 2013-02-27

**Authors:** Elisavet A Papageorgiou, Philippos C Patsalis

**Affiliations:** 1 NIPD Genetics Ltd, PO Box 27954, 2434, Nicosia, Cyprus; 2 The Cyprus Institute of Neurology and Genetics, PO Box 23462, 1683, Nicosia, Cyprus

**Keywords:** Fetal whole genome recovery, next generation sequencing, noninvasive prenatal diagnosis

## Abstract

Noninvasive prenatal diagnosis of chromosomal aneuploidies, although challenging, has been achieved through the implementation of novel methodologies such as methylated DNA immunoprecipitation and next generation sequencing technologies. Nevertheless, additional developments are required towards the interpretation of other fetal abnormalities of higher complexity, such as *de novo *mutations including microdeletion and microduplication syndromes as well as complex diseases. The application of next generation sequencing technologies towards fetal whole genome recovery has demonstrated great potential to achieve the above goal. In a research article published in *Genome Medicine*, Chen *et al. *presented a novel approach that allowed more robust and accurate characterization of parental alleles compared with previous studies. This was achieved through a revolutionary strategy based on the use of trios and unrelated individuals that simultaneously targets the interpretation of the fetal haplotype and phenotype in one step. It is hereby shown that the implementation of a more accurate experimental design in combination with proper analytical tools can provide robust noninvasive fetal whole genome recovery with the potential for further developments beyond the DNA level.

## Introduction

The implementation of noninvasive prenatal diagnosis (NIPD) for chromosomal aneuploidies has been one of the most fascinating achievements for the scientific world because it can offer a safer diagnosis for the fetus after just 10 weeks of gestation [1-7]. Although the challenges associated with the development of NIPD for common aneuploidies have been overcome, the field of NIPD lies beyond the need for prenatal diagnosis of common aneuploidies. Further expansion is required towards the exploitation and diagnosis of other fetal abnormalities that are subtle, infrequent and are not restricted to copy number changes. The implementation of next generation sequencing technologies in the development of NIPD has revolutionized the field and has provided new insight towards further expansion of NIPD. In 2010, the possibility of using next generation sequencing technologies towards whole genome fetal recovery was described for the first time [8]. More recent studies have supported the initial finding, providing new perspectives in the field of NIPD [9,10].

In a research article published in *Genome Medicine*, Chen *et al. *presented a novel approach to NIPD that allowed more robust and accurate characterization of parental alleles compared with previous studies [11]. We here aim to address the advantages of the methodology applied by Chen *et al. *compared with previously developed methodologies towards noninvasive whole genome fetal recovery. Furthermore, we discuss the potential for future developments of the approach proposed by Chen *et al*.

### Noninvasive prenatal diagnosis for screening fetal abnormalities

The need for further developments towards prenatal diagnosis of other fetal abnormalities, including Mendelian diseases as well as complex diseases, has led to the development of technologies towards noninvasive fetal whole genome recovery. The application of next generation massively parallel sequencing has shown to be a very promising approach that would allow extensive investigation of the fetal genome [8-10]. Nevertheless, the approaches described so far are not free of limitations and challenges, and require further investigation and improvement. Chen *et al. *introduced a novel approach towards noninvasive whole genome fetal recovery that overcomes a large number of limitations associated with previously developed methodologies.

Previous studies have focused on inferring the fetal genotype based on a maternal haplotype-assisted approach with no additional interest in the characterization of the biparental heterozygous sites in the fetal genome. As a consequence, there is an uncertainty regarding the accuracy of paternal allele recovery. Chen *et al. *introduced a novel approach for fetal genome recovery that takes into account the paternal allele transition in maternal plasma [11]. This strategy has allowed the assessment of both fetal genotype and fetal phenotype simultaneously and in one step. They used, for the first time, a combined strategy of trios and unrelated individuals to construct parental haplotypes. This allowed a more robust investigation of the fetal genome compared with previous studies that have used only trios [9,10].

Although previous studies have achieved accurate whole genome fetal recovery, their technology is restricted by the limited cell-free fetal DNA (cffDNA) concentration present in maternal plasma, which allowed only the assessment of cases that had a cffDNA concentration >6% [10]. One of the most important characteristics of the strategy developed by Chen *et al. *is that it allows the reconstruction of the fetal genome irrespective of the cffDNA concentration and sequence depth. This strategy led to 100% maternal and paternal allele recovery in contrast to previous methodologies, which were only able to achieve a recovery rate >91.4% for maternal and 71.60% to 72.94% for paternal alleles [9,10]. This is the first time that such a high recovery rate has been achieved, demonstrating the importance and the need for a combined genotype-haplotype approach, as the haplotype information is essential for complex diseases screening and identification of fetal *de novo *copy number variations, including microdeletion and microduplication syndromes.

#### Analysis pipeline

It is of great interest to evaluate the analysis pipeline applied by Chen *et al. *compared with that applied by other groups. Previous studies have implemented a site-by-site strategy to determine the paternal-specific allele [10], occasionally in combination with population-scale sequencing data [9]. However, the above approaches retrieved only 60.3% to 70% of paternal-specific alleles. These results impose the inaccuracy of using only a site-by-site strategy approach in minute amounts of cffDNA and in low sequencing read depth.

Chen *et al. *have revolutionized the field by introducing a new strategy in which a combination of analytical tools were applied to reveal both the haplotype and genotype of the fetus, in contrast to previous studies that focus on the fetal genotype inference [11]. Fetal haplotype recovery was achieved by using the parental haplotypes obtained by the genetic analysis software BEAGLE (a software package for analysis of large-scale genetic data sets with hundreds of thousands of markers genotyped on thousands of samples) for both parental genotype and grandparents' genotyping data in combination with data obtained from the trio study of the Han Chinese 1000 Genomes Project (pilot II) [12]. They managed to achieve fetal whole genome recovery in one step, with 100% recovery of the paternal allele and characterization of recombination breakpoints, by using the site-by-site strategy approach along with the Hidden Markov Mode and Viterbi algorithm.

### Future directions and conclusions

The strategy developed by Chen *et al. *aimed to recover both fetal haplotype and genotype in one step. They used, for the first time, trios in combination with unrelated individuals and achieved a more accurate characterization of parental haplotypes, reaching a predicted rate of 100%. It is clear that this novel approach will facilitate a broader scope in the field of NIPD, including the characterization of Mendelian diseases, the screening of complex diseases and the identification of *de novo *copy number changes, including microdeletion and microduplication syndromes. Although further developments are still required for more accurate fetal *de novo *mutation detection, the Chen *et al. *study has demonstrated the dynamics associated with the use of three generations of a family, which can potentially lead to the more robust identification of both rare and common mutations in the fetal genome.

Moreover, this study demonstrated an increase in accuracy, especially for paternal allele recovery, even in cases with a limited amount of cffDNA (>6%) and at lower sequence depth. This information is of great importance because the technology will not be limited to early gestation, in contrast to previously developed methodologies.

Accurate and robust noninvasive fetal whole genome recovery will also offer solutions at different levels, such as the DNA level (for example, microdeletions, microduplications and point mutations), the level of epigenetic modifications (for example, methylation and chromatin) as well as the translational level (for example, mRNA and microRNAs). Nevertheless, further experimental designs and analytical tool developments are required to decipher such complex information.

Although the current cost for next generation sequencing - especially at the magnitude of whole genome recovery, - is restrictive, it is expected that further technology developments will reduce the cost and will provide more applicable approaches with short turnaround times for noninvasive fetal whole genome recovery.

## Competing interests

PCP and EAP have filled a Patent Cooperation Treaty patent application for a novel NIPD approach for Down syndrome (PCT Patent Application No. PCT/1B2011/000217) and have extensive experience in the field of NIPD. PCP and EAP are co-founders of NIPD Genetics Ltd in Cyprus. The company aims to commercialize a MeDIP real-time qPCR-based approach that PCP and EAP have developed for the NIPD of Down syndrome.

## Authors' contributions

EAP prepared the manuscript and PCP contributed to the final revisions. Both authors read and approved the final manuscript.

## Authors' information

PCP is the Chief Executive Medical Director of the Cyprus Institute of Neurology and Genetics, Cyprus. EAP is the Chief Scientific Officer at NIPD Genetics Ltd. and a Visiting Scientist of the Cyprus Institute of Neurology and Genetics.

## Pre-publication history

The pre-publication history for this paper can be accessed here:

http://www.biomedcentral.com/1741-7015/11/56/prepub
